# Anti-*DEspR* antibody treatment improves survival and reduces neurologic deficits in a hypertensive, spontaneous intracerebral hemorrhage (hsICH) rat model

**DOI:** 10.1038/s41598-023-28149-3

**Published:** 2023-02-15

**Authors:** Victoria L. M. Herrera, Christopher M. Gromisch, Julius L. Decano, Khristine Amber Pasion, Glaiza L. A. Tan, Ning Hua, Courtney E. Takahashi, David M. Greer, Nelson Ruiz-Opazo

**Affiliations:** 1 Whitaker Cardiovascular Institute, Boston University Chobanian and Avedisian School of Medicine, Boston, USA; 2 Department of Radiology, Boston University Chobanian and Avedisian School of Medicine, Boston Medical Center, Boston, USA; 3 Department of Neurology, Boston University Chobanian and Avedisian School of Medicine, Boston Medical Center, Boston, USA

**Keywords:** Molecular medicine, Stroke

## Abstract

Progressive secondary brain injury—induced by dysregulated neuroinflammation in spontaneous intracerebral hemorrhage (sICH)—underlies high sICH-mortality and remains without FDA-approved pharmacotherapy. Clinical insight that hematoma-directed interventions do not improve mortality prioritizes resolving acute secondary brain injury in sICH. As neutrophils are implicated in sICH secondary brain injury, we tested whether inhibition of a rogue neutrophil-subset expressing the dual endothelin-1/signal peptide receptor (DEspR) and associated with secondary tissue injury, DEspR+ CD11b+ immunotype, will attenuate mortality in a hypertensive-sICH (hsICH) rat model. We confirmed sICH-related deaths in hsICH-rats by T2*-weighted 9.4 T MRI and DEspR+ neutrophils in hsICH-rat brain perihematomal areas by immunostaining. At acute sICH, anti-DEspR muIgG1-antibody, mu10a3, treatment increased median survival in hsICH rats *vs* controls (p < 0.0001). In pre-stroke sICH, weekly 10a3-treatment did not predispose to infection and delayed sICH-onset *vs* controls (p < 0.0001). As potential sICH-therapeutic, we tested humanized anti-DEspR IgG4^S228P^-mAb, hu6g8. In vitro, hu6g8 reversed delayed-apoptosis in DEspR+ CD11b+ neutrophils. In vivo, hu6g8 increased median survival and reduced neurologic symptoms in male/female hsICH-rats *vs* controls (p < 0.0001). Altogether, preclinical efficacy of inhibition of DEspR+ CD11b+ neutrophils in acute sICH—without infection complications, supports the potential of anti-DEspR therapy in sICH. Data provide basis for clinical study of DEspR+ CD11b+ neutrophil-subset in sICH patients.

## Introduction

Spontaneous intracerebral hemorrhage (sICH) has the highest mortality among all stroke-subtypes, ranging from 25 to 48% at one month^1^. Poor outcomes reflect the lack of FDA-approved pharmaco/biologic therapies despite much research, and the multifactorial complexity of sICH pathogenesis and progression. Low translatability of past preclinical studies shows the pathophysiological limitations of animal modeling of ICH secondary brain injury via injection of autologous blood or collagenase into normotensive animal brains, and the shortfall of preclinical study endpoints that do not include survival as an endpoint^2^, a key FDA-required clinical trial primary endpoint. In clinical trials, reduction of severe hypertension at sICH onset (the frequent precipitating event) and hematomal evacuation (the primary injury) have not decreased sICH mortality^3,4^. These observations reiterate the dual need to resolve both primary and secondary brain injury mechanisms to reduce early sICH-related deaths^2^, as perihematomal edema and neuronal toxicity can progress despite hematomal stabilization.

However, multiple approaches aimed at reducing secondary brain injury in ICH—whether via inhibition of neuroinflammation, neutrophil activation or adhesion, complement activation, iron-induced oxidative damage, neuronal apoptosis, matrix degradation, glutamate excitotoxicity, and endothelial dysfunction—have not reduced short-term mortality in sICH patients despite preclinical efficacy^2^. Collective reassessment of observed low translatability to the clinic point to the need for preclinical efficacy using sICH animal models that better recapitulate sICH pathogenic risk factors, spontaneous ICH-onset with neurologic deficits, secondary brain injury progression to high mortality or low recovery, and with reasonable logistic feasibility that allows the use of survival as an endpoint in order to improve translatability^5^. Equally important, these clinical trial shortfalls also indicate the need to find novel therapeutic targets involved in secondary brain injury progression to sICH-related death.

Neutrophils have been implicated as drivers of neuroinflammation-mediated secondary brain injury in sICH, as have activated microglia and monocyte-macrophages^6^. However, the association of an elevated peripheral neutrophil-to-lymphocyte ratio (NLR) with poor outcome and high mortality in sICH^7,8^ points to a central role of neutrophil-mediated secondary brain injury in sICH. In this pathophysiological context, and given emerging neutrophil heterogeneity^9^, a putative dysregulation-prone neutrophil-subset with delayed apoptosis likely functions as a “gateway switch” between immediate sICH-induced reaction in the brain and a consequential pro-*injury* inflammatory response leading to secondary brain injury, rather than a pro-*resolution* inflammatory response. In support of this, the majority of the cytokines elevated in sICH patients and associated with worse intraventricular hemorrhage and perihematomal volumes^10^ or mortality^11^, affect neutrophils directly or indirectly.

This notion is also supported by our recent identification of a DEspR+ CD11b+ activated neutrophil-subset with delayed apoptosis that is associated with severity and mortality in patients with acute respiratory distress syndrome (ARDS) progressing to multi-organ failure^12^. This notion is also supported by our chance observation that intravenous infusion of the anti-DEspR prototype mAb, 10a3, during molecular imaging of DEspR+ pathological vasa vasorum angiogenesis^13^, averted progression to death in a Dahl Salt-sensitive hypertensive-spontaneous ICH (hsICH) rat. The hsICH rat model presents with a spectrum of acute neurological symptoms that progress to ICH-related death, presents earlier in females and is worsened by hyperlipidemia^14^. As reported, hsICH rats exhibit severe hypertension (SBP ≥ 200 mmHg) by 3 months of age, increased activated CD11b+ neutrophils, elevated plasma interleukin-18 levels^14^, and differential proteomic changes in brain microvessels in the pre-sICH stage compared to non-sICH age-matched rats^15^.

We therefore tested the hypothesis that anti-DEspR antibody therapy at acute sICH will improve median survival in hsICH rats. As a corollary, anti-DEspR efficacy without anti-hypertensive therapy would confirm the pathogenic role of neutrophil-mediated secondary brain injury in neurological deterioration and progression to ICH-related death. Here we show preclinical efficacy of anti-DEspR therapy and concomitant safety in the acute and pre-sICH stage in the hsICH rat model. Data also validate the humanized anti-DEspR IgG4^S228P^ antibody as a candidate biologic therapeutic for sICH using the hsICH rat model. Altogether, data provide evidence to perform clinical studies in sICH patients testing whether DEspR+ neutrophils correlate with severity and poor outcomes in sICH patients, setting the stage for future clinical trials.

## Methods

### Overall animal model study design

All animal studies comply with ARRIVE guidelines and with our IACUC-approved animal-use protocol with USDA category-E approval for sICH treatment efficacy studies. Animals were assigned to study groups distributing litter and sex representation. Blinded experimental set-up was enforced: lab animal science and veterinary staff observers detecting neurologic deficits at acute sICH or end-stage determinants for euthanasia were blinded to study groups. Neurological symptoms were video-recorded for blinded objective review and post hoc grading of neuro-deficit scores. Treatment was given to hsICH-rats at acute sICH alternating between control and treated rats while establishing equivalent numbers of rats from different litters for each group as the study progressed.

### Hypertensive, spontaneous intracerebral hemorrhage (hsICH) rat model

We used the hypertensive, spontaneous intracerebral hemorrhage (hsICH)-rat model, the Dahl salt-sensitive (S) rat strain made sICH-prone via gestational exposure to Purina 5001 0.4% NaCl rat chow, instead of the Dahl S rat maintenance diet of 0.23% NaCl, and documented for reproducible sICH progressing to early sICH-related death phenotype either as hyperlipidemic (transgenic for human cholesteryl ester transfer protein or CETP) or normolipidemic (non-transgenic)^13–16^. The transgenic hCETP Dahl S rat line is cryogenically preserved in the NIH Rat Resource and Research Repository (NIH P40 OD011062, Columbia, MO).

### Immunofluorescence staining of hsICH-rat brain sections

Immunofluorescence staining was performed as previously described^13,17^. Briefly, fixed, paraffin-embedded sections were processed for immunostaining after antigen-retrieval. Double immunofluorescence staining used: rat-specific anti-DEspR mAb (10a3) labeled with AlexaFluor(AF)-568, 100 μg/ml, and anti-alpha-smooth muscle actin, αSMA (Sigma Aldrich, MO) per manufacturer’s specifications. Primary antibody incubations were done overnight at 4 °C, protected from light. Immunohistochemistry-DAB was done for staining of glial fibrillary acid protein (GFAP) using anti-GFAP GA-5 antibody (sc-58766, Sta. Cruz Biotechnology).

### Testing efficacy/safety of anti-DEspR 10a3 mAb at acute sICH

Treatment followed after at least 1-h of observation with video-documentation of non-transient or progressive neurological deficits. hsICH rats were given a single intravenous (iv) dose of anti-DEspR monoclonal antibody (mAb), 10a3 at 50 μg/kg in 200 μl saline via tail vein under isoflurane anesthesia. For controls, isotype-mock-treated (dose-matched) or non-treated (isoflurane control) rats were used. Rats were monitored 6x/day (weekdays), 3x/day (weekends) and given supportive care (easy food/water access, or intraperitoneal normal saline injection to prevent dehydration). No other therapies were given. Non-responders were euthanized per study endpoints approved by the IACUC and brains examined post-mortem.

### Testing efficacy/safety of multi-dose anti-DEspR 10a3 during pre-sICH stage

To identify the imminent pre-sICH stage, we waited for the first sICH-event (“signal rat”) among a multi-litter cohort of closely age-matched female sICH rats, randomly assigned to treatment (10a3 mAb at 1 mg/kg/dose/week iv × 4-weeks) or control non-treated group. We used non-treated control rats to eliminate inadvertent premature death from isoflurane anesthesia as a confounder in the pre-sICH rat cohort monitoring for infection risk and timing of onset of sICH. Rats were monitored daily for neurologic deficits as described above, as well as for potential adverse effects and robust signs of common infections when risk increases: diarrhea, pneumonia, skin lesions, conjunctivitis.

### Testing efficacy/safety of humanized anti-DEspR IgG4^S228P^ mAb, hu6g8 after acute sICH

The identical protocol used for 10a3 (described above) was followed to test hu6g8 in the acute sICH stage: 3 mg/kg/dose IV × 1 dose after documentation of sICH neurological deficits in both female and males, in both hyperlipidemic transgenic + and wildtype (non-transgenic) hsICH rats. Controls comprised non-treated hsICH rats to eliminate premature deaths from isoflurane anesthesia. Rat brains were collected at end stage for post-mortem analysis, photographed then placed in 4% PBS-buffered paraformaldehyde. Ex vivo 9.4 T MR-Imaging was then performed on fixed hsICH rat brains.

### Isolation of membrane bound-anti-DEspR mAb and non-membrane bound proteins

To measure target engagement of anti-DEspR mAb on DEspR+ neutrophils transmigrating through the blood brain barrier or infiltrated into the brain, membrane bound proteins were isolated. Briefly, rat whole brains were collected and weighed after the animal was perfused with 50 ml ice-cold phosphate buffered saline (PBS). Brain tissue (2-g) was homogenized in two volumes of homogenization buffer (20 mM Tris–HCl pH 7.4, 250 mM Sucrose, 1 mM EDTA and 1% Protease inhibitor) by using a 10 ml Wheaton glass homogenizer applying 10–15 strokes. The tissue lysate was centrifuged at 11,000×*g* for 10 min at 4 °C.

To measure neutrophil-burden in the brain, we measured myeloperoxidase (MPO) levels predominantly released into the neuroparenchyma by neutrophils in non-membrane-bound brain proteins. After removal of membrane-bound proteins, the post-mitochondrial supernatant protein fraction was collected and the total volume was measured before storing 2 ml-aliquots of this protein fraction in − 80 °C freezer. The remaining pellet (membrane protein fraction) was resuspended in 2 volumes of 0.1 M glycine, pH 2.5 until homogeneous. The lysate was centrifuged at 11,000×*g* for 10 min at 4 °C. The supernatant containing the eluted mAb proteins in the Glycine Membrane (GM) fraction was collected. Two milliliter aliquots of the GM fraction supernatant were transferred to a fresh 15 ml conical tube containing 130 µl of 1 M Tris–HCl pH 9.5. The sample was vortexed for 15 s before storing 500 µl aliquots of this mAb protein fraction in a − 80 °C freezer for future analysis.

### ELISA for MPO and mouse-specific immunoglobulin (IgG)

Individual ELISA protocols were performed as per manufacturer’s instructions. Plasma levels of 6g8 and 10a3 mAbs were determined by using a mouse IgG ELISA kit (LSBio Cat# LS-F10451) (plasma dilution 1:100). Quantification of 6g8 mAb in the glycine membrane fraction (target engagement) was done by using the mouse IgG ELISA kit (LSBio Cat# LS-F10451). Quantification of MPO in the post-mitochondrial supernatant fraction (target bioeffects) was performed using the MPO ELISA kit (Hycult Biotech Cat# HK105) at a 1:10 dilution.

### Flow cytometry analysis of LPS-induced DEspR+ CD11b+ neutrophils

Flow cytometry analysis was done essentially as described^12^. Rats were injected intravenously with fresh LPS (Sigma Cat#L2630) 1.8 mg/kg in saline (final volume of 500 μl) 2 h prior to blood collection. Whole blood was collected (EDTA anti-coagulation): 100 μl per tube, × 2 replicates were processed for flow cytometry immediately. Flow cytometry buffer comprised of Hank’s balanced salt solution (HBSS, Invitrogen, NY) plus 2% heat-inactivated FBS as blocking agent; staining antibodies: 10 μg/ml of AF-568 labeled hu6g8 mAb, or the corresponding human IgG4-AF568 isotype-control, and 2.5 μg/ml anti-CD11b-AF488 (Invitrogen, cat#11-0118-42) or the corresponding mouse IgG1 kappa isotype-AF488 control (ThermoFisher Sci. cat# 53-4714-42)^12^. Replicate samples were incubated for 30 min at 4 °C in the dark rotating, then washed with 1 ml FCM-buffer, resuspended in 100 μl 1X PBS, and fixed by adding 100 μl PBS-buffered 2% PFA pH7.4 (for 1% final) for 10 min on ice protected from light, after which the fixative was washed off with 1 ml HBSS, 2% FBS, spun, then cell pellet was resuspended in 2 ml of 1 × lysis buffer (BioLegend RBC Lysis Buffer, cat# 42,031), incubated for 10 min at room temperature (RT), spun, then resuspended in 400 μl HBSS 2% FBS, filtered and analyzed on a BD LSR-II flow cytometer (BD Biosciences), and subsequently analyzed using FloJo Flow Cytometry Analysis Software (www.FloJo.com)^12^

### Ex vivo 9.4 T MRI analysis of hsICH rat brains

Ex vivo MRI scans of fixed hsICH rat brains were acquired at a 9.4 T preclinical MRI scanner (Bruker BioSpec 94/30 system). Multi-echo T2-images were acquired using a multi-slice-multi-echo sequence (MSME). Scan parameters were: repetition time (TR) = 7000 ms, echo spacing = 8.31 ms. The images of 2nd echo from MSME sequence were selected to represent the T2-weighted images. Multi-echo T2* images were acquired using multi-gradient-echo sequence (MGE). Scan parameters were: TR = 6090.75 ms, TE = 5.72 ms, echo spacing = 8.92 ms. The shared parameters for both MSME and MGE were: echo number = 12, FOV = 16.8 × 10.8 cm^2^, matrix size = 252 × 162, slice thickness = 0.5 mm. The images of the second echo from MGE sequence were selected to represent the T2*-weighted images.

### In vitro analysis of DEspR-inhibition effects on survival of in vivo LPS-stimulated rat neutrophils

Rats were injected intravenously with fresh LPS (Sigma Cat#L2630) 1.8 mg/kg in saline (final volume of 500 μl) 2 h prior to blood collection. Five mls of whole blood were collected in an EDTA collection tube (BD Biosciences Cat#366450) and diluted with 5 ml of Cell-based Assay Red Blood Cell lysis buffer (Cayman Cat#600611), mixed and layered over 3 ml of Histopaque (Sigma Cat#11191) in a 15 ml Falcon tube. The sample was spun 500 g for 30 min at RT. The yellow and clear top layer were discarded and the red pellet containing the RBCs and neutrophils were resuspended in 10 ml of Cell-based Assay Red Blood Cell lysis buffer (Cayman Cat#600611) and incubated for 10 min at RT to lyse the RBCs. Neutrophils were collected by centrifugation at 1200*g* × 10 min, washed with 5 ml RPMI-1640 medium (Sigma Cat# R0883) containing 1% BSA and resuspended in 2 ml RPMI-1640 containing 1% BSA. For the inhibition of survival assay, 50,000 live cells in 200 µl RPMI-1640 containing 1% BSA were seeded in P96 well plates. Each condition was performed in 4 replicates testing increasing concentrations of mAb (0.3–30 μg/ml). After 6 h at 37 °C, 5% CO_2_ incubation, live and dead cells were counted by using trypan blue vital stain.

### Rat adapted, modified Rankin Scale (mRS) for neurologic deficit score (NDS)

Because sICH occurs in different brain regions in the hsICH-rat model, we adapted the clinical modified Rankin scale (0–6) for rats into the NDS which allows rat-modified mRS grading without need for task-specific behavioral testing, and allows assessment of severity of neurological deficits via respective or collective impact on normal rat activity.

Neurologic Deficit Score:

0: No symptoms.

1: Neurological deficit present but no significant disability. Rat is able to carry out all usual activities: walk, feed, drink, groom. Intermittent focal symptoms, or persistent mild deficits.

2: Slight disability, unable to carry out all previous activities because of more frequent neurological symptoms leading to less activity, but does not require help.

3: Moderate disability. Rat requires some help (food access) due to neurological deficits, but able to walk unassisted.

4: Moderately severe disability. Due to neurological deficits, the rat is unable to attend to own bodily needs without assistance, and/or unable to walk unassisted; and with intermittent decreased level of consciousness is detected.

5: Severe disability. Requires care, rat unconscious, does not move when touched; no cardio-respiratory distress. Hydration provided.

6: Dead, or positive cardio-respiratory euthanasia endpoint.

### Radiotelemetric implantation for non-stress measurement of blood pressure and activity

To measure effects of anti-DEspR hu6g8 antibody on blood pressure, intra-aortic abdominal radiotelemetric implants (DATASCIENCE, St. Paul, MN) were surgically implanted under anesthesia as described^18^, in naïve 4 m-old Dahl salt-sensitive (S) female rats. After 1-week from procedure and complete wound healing, blood pressure readings were obtained taking the average over 10 s every 5 min for 24 h, 288 datapoints/day. Telemetric data were collected for systolic and diastolic blood pressure (SBP, DBP), mean arterial pressure (MAP), as well as heart rate and activity for 3 consecutive days before treatment, followed by 2-day rest period, then treatment with 10a3 (50 μg/kg, 1 dose via tail vein) and telemetric blood pressure datapoints collected for 3 consecutive days. Mean ± SD graphed.

### Statistical analyses

For hsICH-rat model survival studies comparing control vs treated hsICH-rats, we used Kaplan–Meier survival curves testing for statistical significance by using the Mantel Cox Log Rank Sum test and Holm-Sidak multiple comparison testing (GraphPad Prism v9.2, SigmaStat). For control vs antibody-treated group comparisons, we used the two-tailed Mann Whitney test for analysis of significance of differences (GraphPad Prism v9.2), Hedge’s g statistic less 4% correction for effect size: g > 0.8 indicates large effect size (https://www.statisticshowto.com/hedges-g/). Kruskal Wallis ANOVA on ranks and Dunn’s multiple comparison testing was performed for age at sICH-onset comparison (GraphPad Prism v9.2). All data sets conformed to the assumptions of each specific statistical test. P < 0.05 was considered statistically significant.

### Ethics declarations

This study was performed in strict accordance with the recommendations in the Guide for the Care and Use of Laboratory Animals of the National Institutes of Health. The protocol was approved by the Committee on the Ethics of Animal Experiments of Boston University School of Medicine (Permit Number: AN-14055) with Category E approval. Euthanasia of study animals was done by removal of vital organs and exsanguination under general anesthesia as stated in the 2013 AVMA Guidelines.

## Results

To test the hypothesis that anti-DEspR mAb therapy attenuates neutrophil-mediated progression of post-sICH secondary brain injury to ICH-related death, we tested in vivo preclinical efficacy of our murine prototype anti-DEspR monoclonal antibody (mAb), 10a3, while observing for potential adverse effects after acute sICH presentation to determine single-dose effects on progression to early-mortality in the hsICH-rat model^14,15^; and in the pre-ICH stage to assess multi-dose effects on potential infection risk and adverse events in the presence of ongoing sICH pathogenic progression. Addressing translatability, we also tested preclinical efficacy of the humanized anti-DEspR mAb with a hinge-stabilized human IgG4-backbone, hu6g8, after acute sICH.

**Figure 1:**
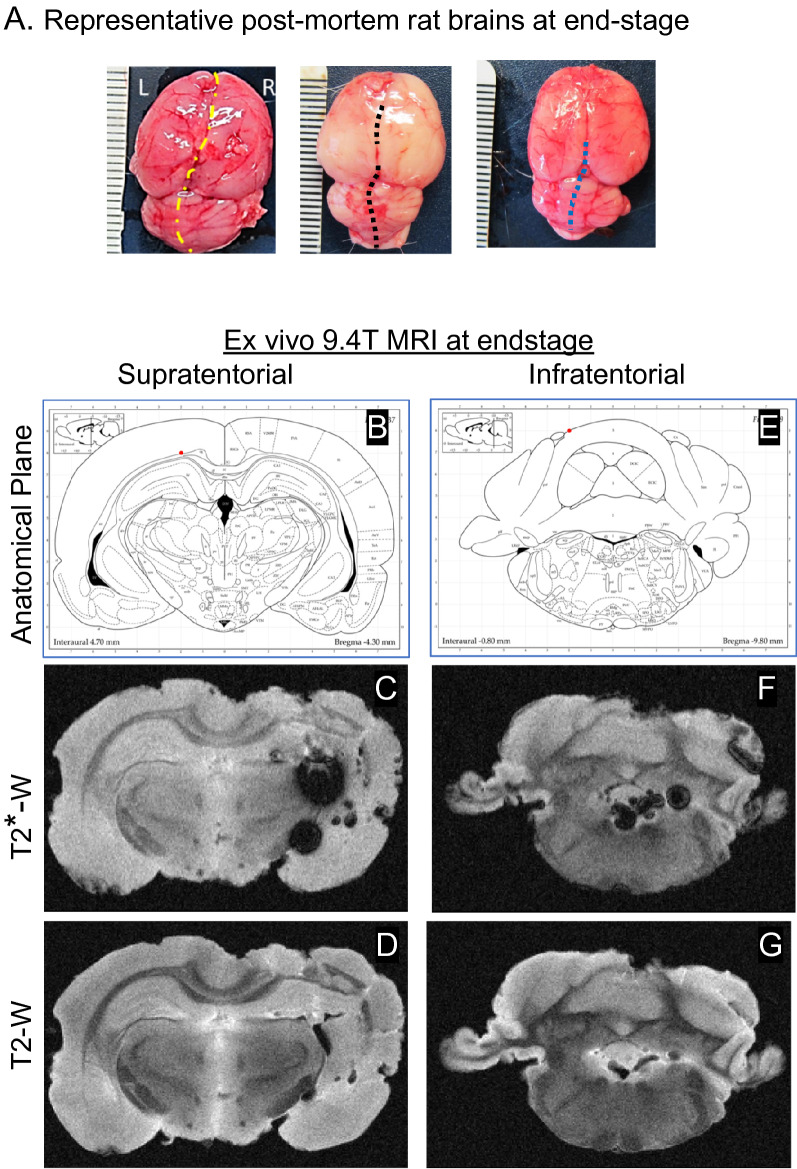
(**A**) Representative post-mortem images of hsICH rat brains isolated immediately after euthanasia depicts hemorrhages. (**B**–**G**) Representative ex vivo 9.4 T MRI of hsICH rat brain after ICH-related death showing sICH phenotype in supratentorial site (**B**–**D**) and infratentorial site (**E**–**G**) in a control rat. (**B**) Diagram of anatomical plane through thalamus, dorsal 3rd ventricle and lateral ventricles. (**C**) T2* weighted (T2*-W) intraventricular hemorrhage (IVH) in the left lateral ventricle. (**D**) T2-weighted (T2-W) image showing areas of perihematomal edema (PHE). (**E**) Diagram of anatomical plane through medulla, cerebellum, 4th ventricle and recess of 4th ventricle. (**F**) T2*-W gradient echo MRI showing IPH in the plane of the cerebellum and medulla, IVH in the 4th ventricle. (**G**) T2-W image in the same plane as (**F**) showing edema and IVH in the 4th ventricle and recess. MRI right, rat left.

We selected the hsICH rat model^14^ as it fulfills key recommendations from the Hemorrhagic Stroke Academia and Industry (HEADs) roundtable to improve translatability of ICH model studies: recapitulation of hypertension comorbidity, spontaneous onset of ICH, clinically relevant neurological symptoms, progression to stroke-related death, reproducible high mortality/low recovery phenotype, and logistic feasibility to use survival as an endpoint^5^. Compliance with HEADS criteria for translatability of ICH models is shown in the Kaplan–Meier survival curve analysis showing the onset of sICH consistently leading to death in both hsICH female and male rats previously characterized^14^ (Supplementary Fig. S1), showing earlier onset of sICH in female rats^14^. Notably, with moderate-severe hypertension, sICH exhibits spontaneous onset marked by a spectrum of clinically relevant neurological deficits—seizures, paresis, abnormal posturing and/or movement, with progression of symptoms and progression to death (Supplementary Tables S1, S2) as previously observed^14,16^. Hypertension is worse in the hsICH rat model, reaching > 160 mmHg by 8 weeks in both female and male rats compared to non-sICH rats^14^. At end-stage, post-mortem analysis of brains shows brain hemorrhages visible on the surface, as well as evidence of brain swelling with distortion of anatomical midline (Fig. 1A).

### Ex vivo 9.4 T MRI confirms ICH-related death in the hsICH rat model

To ascertain the hsICH-phenotype at ICH-related death, we performed ex vivo 9.4 T MRI on fixed rat brains. As shown in Fig. 1, ex vivo 9.4 T MRI showed IPH, perihematomal edema (PHE), and intraventricular hemorrhage (IVH) in supratentorial and infratentorial locations in a representative hsICH female rat brain at end-stage (Fig. 1B–G; see Supplementary Fig. S2), confirming sICH as the cause of death. MRI findings at end-stage are distinguished in severity compared to the acute sICH stage in the hsICH rat model (Supplementary Fig. S2).

### Verification of histopathology and DEspR+ neutrophils in hsICH-rat brain at the acute ICH stage

**Figure 2:**
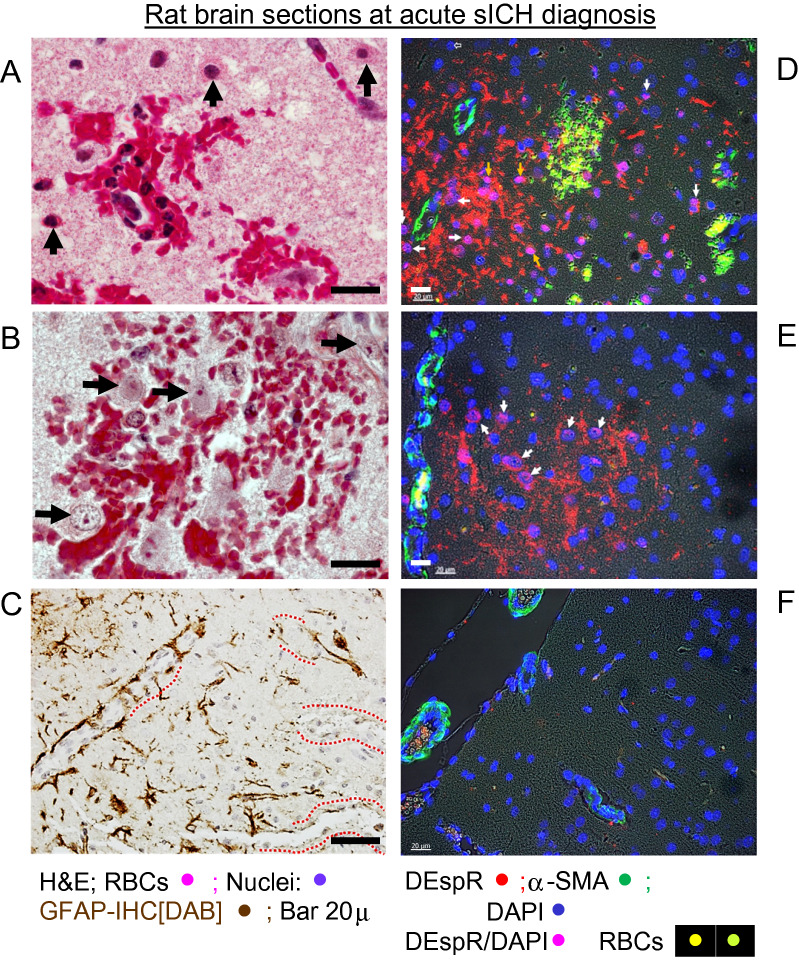
Representative histopathological analysis of rat brain sections at sICH detection. (**A**) H&E staining of microbleed area with neutrophil infiltrates; ↑ pyknotic, eosinophilic neurons. (**B**) H&E staining of microbleed area with no neutrophils: neurons not pyknotic nor eosinophilic. (**C**) Immunohistochemistry-DAB staining of GFAP; dashed red line (**----**) mark microvessels with non-contiguous GFAP immunostaining suggesting BBB disruption. (**D**,**E**) Representative immunofluorescence staining of sICH rat brain sections at acute ICH detection; white arrows → DEspR+ neutrophils examples. Red DEspR+ anuclear fragments noted. (**F**) IF-staining of age-matched non-ICH Dahl S rat brain section. DEspR+ (red); RBCs, red blood cells autofluorescence (yellow-green); αSMA, alpha smooth muscle alpha actin (green) serves as positive-IF control for microvessels; DAPI, nuclear stain; merged DEspR+ DAPI+, magenta. Bar 20 microns.

To document histopathology in peri-hematomal areas in the hsICH rat brain, we performed H&E histological staining. In the peri-hematomal areas with microbleeds, high magnification images detected association of transmigrated neutrophils, red blood cells and pyknotic, eosinophilic neurons indicating dying neurons (Fig. 2A). In contrast, microbleed areas with no neutrophils did not exhibit pyknotic, eosinophilic dying neurons (Fig. 2B). Concordantly, immunohistochemistry staining for glial fibrillary acidic protein (GFAP) showed microvessels with incomplete astrocyte endfeet (Fig. 2C), concordant with the lack of normal endfeet replacement^19^, hence non-contiguous astrocytic endfeet-mediated modulation of endothelial blood brain barrier functions^20^.

Next, we performed immunofluorescence-staining (IF-staining) on fixed, paraffin-embedded brain sections using fluorescently labeled anti-DEspR 10a3-mAb, to verify presence of DEspR+ neutrophil infiltrates in hsICH rat brain on day of acute sICH-presentation. After we verified high affinity binding of 10a3 mAb to the DEspR antigenic peptide used to develop it (Supplementary Fig. S3), fluorescently labeled 10a3-AF568 IF-staining detected DEspR+ neutrophils in peri-hemorrhage areas (Fig. 2D,E), but not in control age-matched non-sICH rat brain section (Fig. 2F). DEspR+ neutrophil nuclei are also observed.

### In vivo efficacy with prototype anti-DEspR 10a3 mAb in the acute sICH-stage

**Figure 3:**
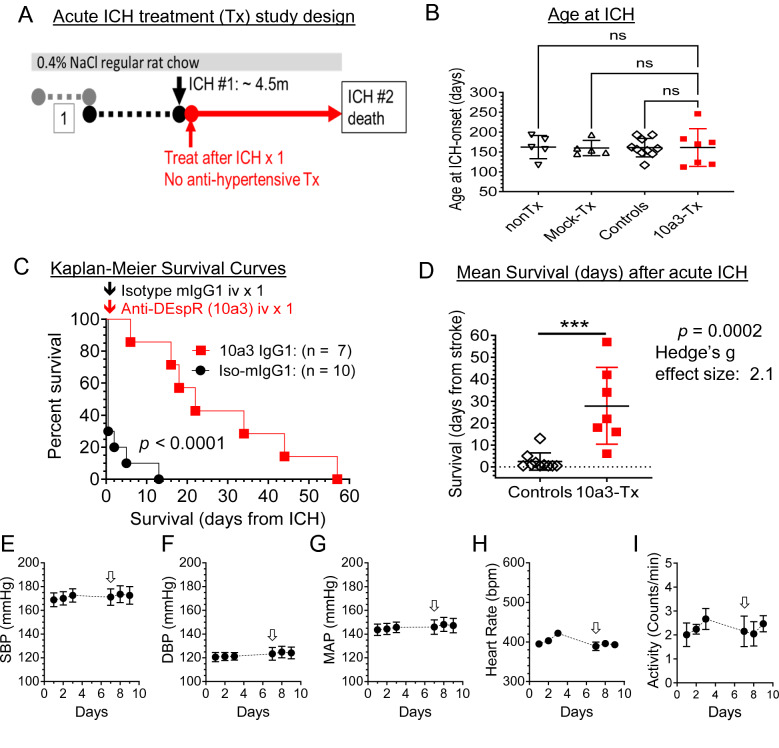
Preclinical efficacy testing of single-dose anti-DEspR murine mAb prototype, 10a3, at acute sICH. (**A**) Diagram of experimental design of onset of treatment (anti-DEspR mAb 10a3, 50 μg/kg/dose × 1) or mock treatment (isotype murine IgG1, 50 μg/kg/dose × 1) given after first ICH (#1) documented. Monitoring until the 2nd stroke with SBP remaining at ≥ 200 mmHg; no anti-hypertensive therapy given. (**B**) Graph of age at onset of sICH among study groups: treated rats (n = 7), total control group (n = 10), made up of non-treated (nonTx, n = 5) and muIgG1-isotype (mock-Tx, n = 5). Kruskall Wallis ANOVA on ranks and Dunn’s multiple pairwise comparison were not-significant. (**C**) Kaplan–Meier Survival curves comparing treated (n = 7) and all control (n = 10) hsICH rats: median overall survival (Control vs Treated: 0.5 *vs* 22 days); Mantel Cox log-rank test p < 0.0001. Log rank hazard ratio 3.5, (95% CI 1.23–10.05). (**D**) Two-tailed unpaired Mann Whitney rank test of survival to 2nd stroke comparing controls (n = 10), mean ± sd: 2.4 ± 3.96) vs treated (n = 7: 28.9 ± 17.5), ***p = 0.0002 with Hedge’s g effect size [− 4%] = 2.1. (**E**–**I**) Average 24-h blood pressure and activity measurements obtained via radio-telemetry implant system before and after single-dose anti-DEspR 10a3 treatment (↓) in naïve 4 m-old Dahl S female rats (n = 6): (**E**) systolic blood pressure, SBP, (**F**) diastolic blood pressure, DPB, (**G**) mean arterial pressure (MAP), (**H**) heart rate, and (**I**) activity (number of times the single-housed rat crosses the midline section of the cage).

Testing for preclinical efficacy of the anti-DEspR antibody to slow secondary brain injury and increase survival, we designed an acute sICH-stage treatment study using the hsICH rat model (Fig. 3A). At around 4.5 months of age, sICH is identified by the onset of acute neurologic deficits ranging from paresis/paralysis to seizures, abnormal movement/gait, posturing or loss of consciousness. After documenting non-transient or progressive neurological deficits by video, anti-DEspR 10a3 muIgG1 antibody intravenously (IV) via tail vein. To demonstrate anti-DEspR effects, antihypertensive therapy was not given for SBP in the ≥ 200 mm Hg, as reported for the hsICH rat model^14^. Control groups comprised of both mock isotype-treated and non-treated hsICH rats. These two control groups ascertain anti-DEspR effects rather than from IgG infusion or from isoflurane anesthesia respectively. The ranges of age at sICH onset among study groups were not statistically different (Fig. 3B).

Kaplan Meier survival curve analysis of treatment in the acute sICH-stage compared with control hsICH rats detected significant differences (p < 0.0001) in median survival to second sICH event (Fig. 3C). All control hsICH rats died after the first sICH event (median 0.5 days), in contrast to 10a3-treated rats, all of which recovered, succumbing only to a second sICH-event which marked the survival endpoint (median 22 days). We note that a second sICH-event was expected, as no anti-hypertensive therapy for SBP ≥ 200 mmHg was given. Analysis of differences in survival after the first sICH-event between treated animals and controls showed statistical significance (p = 0.0002) with a large effect size measured as Hedge’s “g” > 0.8 (Fig. 3D).

To clarify the potential mode-of-action inhibiting neutrophil-mediated secondary injury, radiotelemetric 24/7 blood pressure measurements were obtained in naïve, age-matched 4 month-old Dahl S female rats (Fig. 3E–H). Data showed that anti-DEspR antibody (10a3)-treatment did not reduce systolic, diastolic, or mean blood pressure (Fig. 3E–G), nor heart rate (Fig. 3H). Additionally, radiotelemetric measurement of activity (# of times a rat crosses midsection in its cage) showed that DEspR-inhibition did not induce adverse effects that reduced rat activity (Fig. 3I).

### In vivo study of multi-dose anti-DEspR 10a3 adverse effects and infection risk in pre-sICH

**Figure 4:**
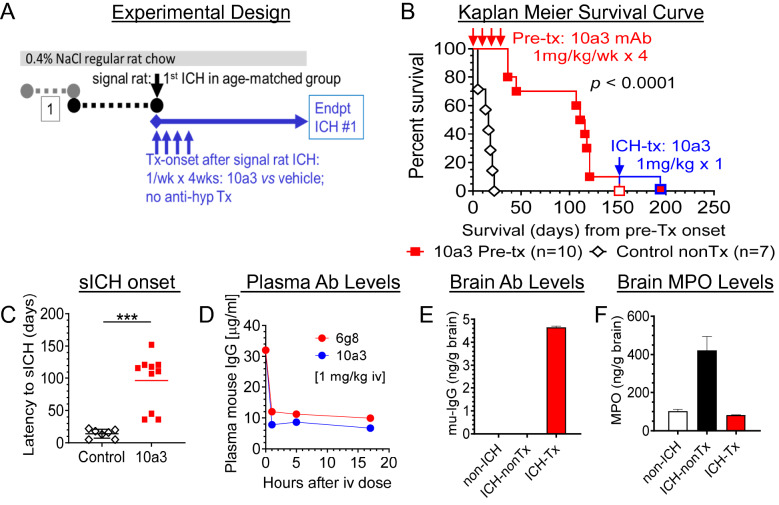
Preclinical testing for potential infection-risk or adverse events using multi-dose anti-DEspR mAb prototype, 10a3, in imminent pre-ICH stage. (**A**) Diagram of experimental design: monitor for 1st sICH-event identifying “signal” rat and onset of imminent pre-sICH stage in age-matched cohort, randomize to treatment or vehicle-control group; start 10a3-treatment: 1 mg/kg/dose iv/week × 4 weeks; daily monitoring; no anti-hypertensive meds. (**B**) Kaplan–Meier curve analysis to onset of 1st stroke comparing controls (n = 7) and age-matched 10a3-treated (n = 10) hsICH female rats: significant differences in time to 1st sICH, Mantel-Cox log rank test p < 0.0001 with log rank hazard ratio (95% CI) 5.46 (1.2–24.4). (**C**) Comparison of sICH-onset from day of signal rat stroke between treated and controls: two tailed, unpaired Mann Whitney rank test: ***p = 0.0001. (**D**) Analysis of plasma levels of single-dose anti-DEspR murine prototype 10a3 mAb and 6g8 mAb (murine precursor for humanized anti-DEspR) in imminent pre-hsICH rats (n = 2 rats/group), each at 1 mg/kg/dose iv. (**E**) Brain target engagement: mouse-specific IgG levels in brain membrane bound proteins (mu-IgG ng/ gram brain) of 6g8 in pre-hsICH rat brain (ICH-Tx) compared with control non-sICH (non-ICH) and control nontreated pre-hsICH (ICH-nonTx) rat brains. (**F**) Brain target bioeffect: reduction of brain levels of released myeloperoxidase (MPO) and MPO + neutrophils in ICH-Tx rat brain, compared with control non-ICH and ICH-nonTx.

To strengthen translatability, we tested for potential infection risk and adverse effects that worsen sICH-mortality as observed in failed clinical trials. We tested multi-dose 10a3 at a higher dose (1 mg/kg/dose/week × 4 weeks) in an age-matched, all-female cohort at a vulnerable imminent pre-sICH stage. We defined the onset of the imminent pre-sICH stage after the first sICH-event in the “signal rat” (Fig. 4A). Comparing 10a3-treated vs vehicle mock-treated rat groups, month-long weekly 10a3-treatment did not lead to any infections in regular housing conditions throughout the observation period, nor to any adverse effects (Fig. 4B). In fact, rather than adverse effects accelerating sICH onset, Kaplan Meier curve analysis showed significant delay in onset of sICH in 10a3-treated rats compared to vehicle mock-treated control hsICH rats, p < 0.0001, (Fig. 4B). Mann Whitney rank confirmed significant differences (p = 0.0001), Fig. 4C. We also observed that all control mock-treated hsICH-female rats exhibited their first sICH event within a 21-day range (Fig. 4B), showing logistic feasibility for preclinical studies. We confirmed plasma levels of 10a3-antibody after a single dose in a contemporaneous but distinct subset of hsICH rats by ELISA, measured 24 h after tail vein infusion of 10a3 (Fig. 4D).

Pertinent to translation to the clinic, we also tested 6g8 mAb, the murine precursor for humanized anti-DEspR mAb, hu6g8, selected for cross-species reactivity to rat, monkey and human DEspR with identical epitope sequence^21^. We found similar plasma levels of 6g8 (Fig. 4D), as well as 6g8-localization in pre-hsICH rat brain membrane-proteins 24 h after infusion in contrast to negative controls: non-ICH and non-treated ICH rat brains (Fig. 4E). To assess 6g8-mAb functionality, we detected decreased neutrophil-associated myeloperoxidase (MPO) levels in hsICH-treated rat brain cytosolic proteins in contrast to control ICH-untreated rats and control non-ICH rats (Fig. 4F), indicating 6g8 target bioeffect and basis to test humanized anti-DEspR hu6g8 in vivo efficacy in the hsICH rat model.

### In vitro and in vivo efficacy of humanized anti-DEspR mAb, hu6g8

**Figure 5:**
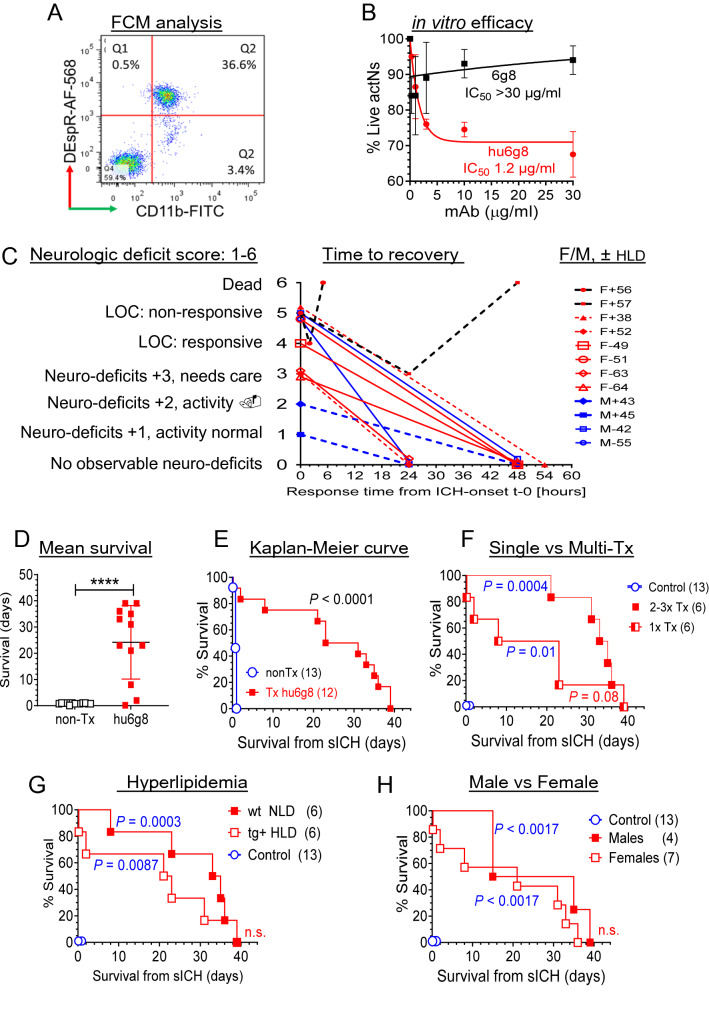
Characterization and preclinical efficacy study of humanized anti-DEspR antibody, hu6g8, in acute sICH. (**A**) Flow cytometry analysis of DEspR+ CD11b+ neutrophils in Dahl S rats induced in vivo upon TLR-4 activation by endotoxin (LPS). Gated DEspR+ /CD11b+ neutrophils depicted in quadrant 2 (Q2). (**B**) Concentration-dependent neutrophil survival assay comparing murine precursor 6g8 and humanized anti-DEspR hu6g8 antibodies. Efficacy defined by decrease in % live neutrophils from untreated controls. Hu6g8 IC_50_ = 1.2 ± 0.3 μg/ml; 6g8 IC_50_ > 30 μg/ml. (**C**) Graph of treated rat profiles: neurologic deficit score before treatment (score 1–6), time to recovery (hours) after single hu6g8 treatment (3 mg/kg iv), sex and whether normal [–] or hyper [+] lipidemic (± HLD). (**D**) Two-tailed Mann Whitney rank test comparing survival (days) of controls non-treated (nonTx, n = 13) and hu6g8-treated (n = 12) p < 0.0001. Hedge’s g effect size [− 4% correction] for difference in means: 2.3. (**E**) Kaplan–Meier survival curve of hu6g8-treated (median overall survival, mOS, 27-days [d]) vs non-treated controls (mOS 0.75d), Mantel Cox Log Rank Sum test *P* < 0.0001, log rank hazard ratio (95% CI) 3.2 (1.3–8.0). (**F**) Kaplan–Meier survival curve Log rank test followed by Holm Sidak multiple comparison testing of hu6g8-treated rats stratified for number of treatments detected *P* = 0.01 for single treatment (mOS 15.5d), *P* = 0.0004 for repeat treatment upon sICH recurrence (mOS 34d); comparison of single vs repeat treatment groups detected *P* = 0.08. (**G**) Kaplan–Meier survival curve Log rank test followed by Holm Sidak multiple comparison testing of hu6g8-treated rats stratified for hyperlipidemia (HLD) : hu6g8-treated HLD + mOS 22d vs non-treated controls 0.75d (n = 13), *P* = 0.0087; hu6g8-treated normolipidemic (NLD) median survival 34d (n = 6) *vs* non-treated controls, *P* = 0.0003; comparison of HDL vs NLD groups was not significant. (**H**) Kaplan–Meier survival curve Log rank test followed by Holm Sidak multiple comparison testing of hu6g8-treated rats stratified for sex detected *P* = 0.0017 for both females (mOS 21d) and males (mOS 25d) respectively compared to control non-treated hsICH rats (mOS 0.75d); comparison of males vs females was not significant.

To enhance translatability by early verification of candidate therapies^5^, we next tested preclinical efficacy of hu6g8. Designed as a recombinant antibody with a hinge-stabilized IgG4^S228P^ backbone to avoid antibody-dependent/complement-dependent cell cytotoxicity (ADCC/CDC)^21^. In stepwise testing of the humanized anti-DEspR mAb, hu6g8, we first verified hu6g8 binding to activated DEspR+ CD11b+ rat neutrophils via flow cytometry (FCM) (Fig. 5A). Next, we verified hu6g8 functionality in inducing apoptosis in DEspR+ CD11b+ neutrophils in a concentration-dependent manner with nanomolar IC_50_ (Fig. 5B).

After in vitro validation, we tested in vivo efficacy of hu6g8 in the acute ICH stage using the experimental model design in Fig. 3A. As best possible, we ascertained distributive representation of litters, sex and similar age at ICH onset to all groups (Supplementary Fig. S4). Study rats spanned the full spectrum of neurological deficits from minimal effects on normal activity to loss of consciousness corresponding to a neurological deficit score (NDS) of 1–5 barring euthanasia indicators (Fig. 5C, Supplementary Tables S1, S2) (see “Methods”). As shown in Fig. 5C–E, without anti-hypertensive therapy, anti-DEspR hu6g8-treatment at acute sICH attenuated neurological deficits resulting in gradual recovery from presenting neurological symptoms to NDS-0 over 1–2.5 days in 10 of 12 sICH rats (Fig. 5C) and non-progression to sICH-related deaths, thus extending median survival compared to control non-treated hsICH rats significantly by Mann Whitney analysis (Fig. 5D), and Kaplan–Meier survival curve analysis (Fig. 5E). Concordant with expected exacerbation of endothelial dysfunction by hypertension and hyperlipidemia leading to increased risk for cerebral microbleeds and sICH recurrence^22^, two hsICH rats with hyperlipidemia did not respond to hu6g8-treatment (Fig. 5C).

With persistent hypertension, recurrent sICH was observed in all treated rats which marked end of life if associated with euthanasia indicators (n = 6 of 12), or if no euthanasia indicators, a 2nd treatment course was given (n = 6 of 12 treated group). Importantly, longer median survival was attained with hu6g8-treatment upon recurrence of acute sICH (NDS 1–5) without euthanasia indicators (Fig. 5F). Notably, both single treatment and multi-treated hsICH rat subgroups exhibited significant differences in median survival from control non-treated hsICH rats respectively (Fig. 5F). Similarly, treated normolipidemic and hyperlipidemic hsICH rats exhibited significant increased median survival compared to control non-treated hsICH rats at acute sICH, respectively, but not significantly different from each other (Fig. 5G). Likewise, female and male hsICH rats showed significant equivalent response with increased median survival over control rats respectively (Fig. 5H).

**Figure 6:**
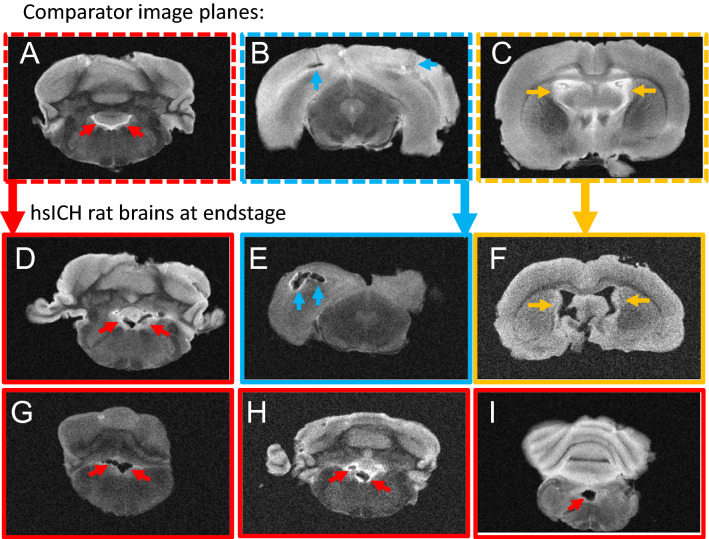
Ex vivo T2-weighted 9.4 T MR-images of hsICH rat brains at end-stage. (**A**–**C**) Representative comparator 9.4 T MR-images at the (**A**) plane of medulla with no blood in the 4th ventricle ( →), CSF shows white on T2-W MRI; (**B**) at the plane of the midbrain showing smaller IPH and PHE; and (**C**) at the plane of CSF-filled (white) lateral ventricles ( →) and 3rd ventricle; no IVH. (**D**–**I**) Representative hsICH rat brains (n = 6) obtained after sICH-death confirming sICH as cause of death showing (**D**) IVH in the 4th ventricle (red →), (**E**) severe IPH with PHE (blue →), (**F**) IVH in lateral and 3rd ventricles (yellow →), (**G**–**I**) IVH in the 4th ventricles (red →). Panels (**A**–**C**) show comparator MR-images for Panels (**D**–**I**) as marked and outlined per color.

As confirmation, post-mortem inspection of rat brains—treated and control groups—exhibited evidence of a hemorrhagic event (Fig. 1A). Concordantly, ex vivo 9.4 T MRI of sICH-rat brains confirmed sICH-related deaths with more extensive IPH, PHE and IVH, including subtentorial IPH and IVH on ex vivo 9.4 T T2-weighted MRI (Figs. 1C–G, 6, Supplementary Fig. S2A), compared with less severe findings on day of acute sICH-presentation (Supplementary Fig. S2B) and as previously observed^14^.

## Discussion

The demonstration of efficacy and safety, in a hypertensive-spontaneous ICH rat model in acute-sICH and pre-sICH stages, altogether show that anti-DEspR mAb therapy has clinically relevant potential to attenuate neutrophil-mediated secondary brain injury in sICH. Implementing experimental designs focused on de-risking translational hurdles learned from clinical trial failures of other candidate therapies for secondary brain injury in sICH or in ischemic stroke despite preclinical efficacy^2^ support the translatability of our findings.

Three distinct in vivo studies using the hsICH rat model presented here confirm that the hsICH-rat model fulfills key recommendations by the Hemorrhagic Stroke Academic Industry Roundtable (HEADS) to improve translatability^5^. While acknowledging species differences, similarities with clinical ICH documented by ex vivo 9.4 T MRI detecting supra- and infra-tentorial ICH with PHE and IVH at end-stage further validate the hsICH-rat model. The detection of extensive IVH at end-stage and not in the acute sICH stage, coupled with delay in sICH-death by anti-DEspR antibody-therapy suggest the hypothesis that DEspR-inhibition of DEspR+ CD11b+ neutrophils at acute-sICH might be a potential “gateway switch” to attenuate progression to IVH-extension. The progressive neurological deterioration toward early death within a week from onset in most controls, parallels clinical observations of PHE development and association with clinical deterioration^23^, leading to early death in severe sICH patients^24^. These clinically relevant similarities support the translatability of findings using the hsICH-rat model.

Using survival as endpoint, studies in the hsICH-rat model are differentiated from studies done in the autologous blood injection-induced ICH mouse/rat model (ABI-iICH) which exhibits focal neurological deficits that typically resolve spontaneously in animals surviving the injection procedure^25^. The differences in high-mortality progression between hypertensive sICH-rat model and ABI-iICH model in normotensive mice/rats highlights the pathogenic significance of hypertension-induced neutrophil-priming and brain endothelial activation present in hsICH-rats, but not in ABI-iICH normotensive animal models^25,26^.

Furthermore, unlike ABI-iICH modeling with precise determination of time of onset^26^, testing in the hsICH rat model evaluates efficacy in the presence of variable latency periods from last “known”/observed-well, and varied severity at treatment. This “take-all-comers” experimental design recapitulates the variable clinical^1,2^ scenarios in sICH patients, and suggests that anti-DEspR therapy efficacy in this context projects a potential clinically feasible therapeutic window. These observations address the lesson from the non-clinically feasible therapeutic window for rovelizumab (anti-CD18 huIgG4-mab) resulting in clinical trial termination for futility in reducing mortality in ischemic stroke^27^, in contrast to rovelizumab’s preclinical efficacy in a rabbit model when rovelizumab is infused 20-min after middle cerebral artery occlusion^28^.

Importantly, anti-DEspR efficacy in the acute sICH-stage with gradual recovery from neurologic symptoms, even in non-responsive rats (NDS 5)—albeit to lesser degree than rats with less severity at treatment onset (NDS ≤ 3)—suggests the potential impact of timely inhibition of secondary brain injury in sICH prior to the point of irreversibility. Further studies are needed to test the potential for greater efficacy when combined with hypertension reduction, as our efficacy studies were done without antihypertensive therapy, and anti-DEspR treatment did not lower SBP, DBP or mean arterial pressure. Additionally, the observed efficacy in recurrent acute hsICH, in both hyperlipidemic and normolipidemic rats, and males and female rats support a common pathogenic event and potential generalizable efficacy in attenuating neutrophil-mediated secondary brain injury in acute sICH.

These observations are concordant with neutrophil-subset specific induction of apoptosis by anti-DEspR mAb in dysregulated “rogue” DEspR+ CD11b+ neutrophils^12^ provides a therapeutic hypothesis for reducing neutrophil-mediated secondary brain injury, as neutrophil apoptosis is required for neutrophil function shutdown, clearance, and initiation of resolution of injury-triggered inflammation^29^. Importantly, the DEspR-targeted approach would result in DEspR+ neutrophil subset-specific apoptosis-mediated function shutdown, while sparing [a] DEspR(−) neutrophil subsets needed for pathogen-defense, [b] DEspR(−) monocyte-macrophages and microglia needed for neutrophil efferocytosis/phagocytosis^30^—all critical to resolution of neuroinflammation^31^.

Most importantly, the safety of anti-DEspR therapy in the acute stage and in the pre-stroke stage without increasing infection risk assuages the inherent fear of increased infection when neutrophils are inhibited. This reiterates the importance of identifying culprit or “rogue” neutrophil-subsets for subset-specific inhibition given neutrophil heterogeneity, rather than global anti-inflammatory glucocorticoids which failed to reduce sICH mortality^2^. Additionally, both anti-DEspR antibodies: 10a3-muIgG1 and hu6g8-IgG4^S228P^—did not induce adverse events from complement-mediated cell cytotoxicity. This is a notable de-risking observation, as sICH is associated with elevated complement activation in the brain, which also contributes to secondary brain injury directly and/or through neuroinflammation^32,33^.

### Limitations of the study

The preclinical efficacy study was limited to post hoc ex vivo MRI of fixed rat brains. We were unable to do serial MRI, nor blood sampling due to the precarious states of hsICH rats at acute sICH which precluded the use of isoflurane anesthesia during MR-imaging and blood draw. The high risk of isoflurane-related death would have confounded all survival-as-endpoint studies. While the NDS gives insight into severity at treatment onset, we were unable to stratify based on hematoma or PHE volumes prior to treatment due to logistic limitations that confound efficacy survival studies.

### Interpretation

Altogether, our study demonstrates the preclinical efficacy and safety of anti-DEspR antibody therapy tested in the hsICH rat model which complies with HEADS-2018 recommendations to improve translatability^5^. The observed effect size on mortality and neurologic symptoms seen with therapeutic inhibition of DEspR+ CD11b+ neutrophil-subset in the hsICH-rat model suggests a potential gateway-role for DEspR+ CD11b+ rogue neutrophils in neuroinflammation. When elevated beyond a putative threshold, DEspR+ CD11b+ neutrophils mediate progression to secondary brain injury. When decreased by therapy below that threshold, homeostatic resolution of inflammation proceeds, attenuating secondary brain injury. Data also validate the humanized anti-DEspR antibody as a potential candidate therapeutic for sICH and provide foundational bases to study dysregulated DEspR+ CD11b+ neutrophils as a potential stratification biomarker and “gateway therapeutic target” in sICH patients, concordant with HEADS-2 recommendations^34^.

## Supplementary Information

Supplementary Information 1.

Supplementary Information 2.

Supplementary Information 3.

## Data Availability

The data analyzed during the current study are available from the corresponding author on reasonable request.
