# Supplementary material for: Anti-DEspR antibody treatment improves survival and reduces neurologic deficits in a hypertensive, spontaneous intracerebral hemorrhage (hsICH) rat model
Source: Sci Rep. 2023 Feb 15;13:2703. doi: 10.1038/s41598-023-28149-3 (PMC9932093; doi:10.1038/s41598-023-28149-3)

Supplementary Fig. S1. Kaplan-Meier Survival Curves of sICH Dahl S rat model subsets

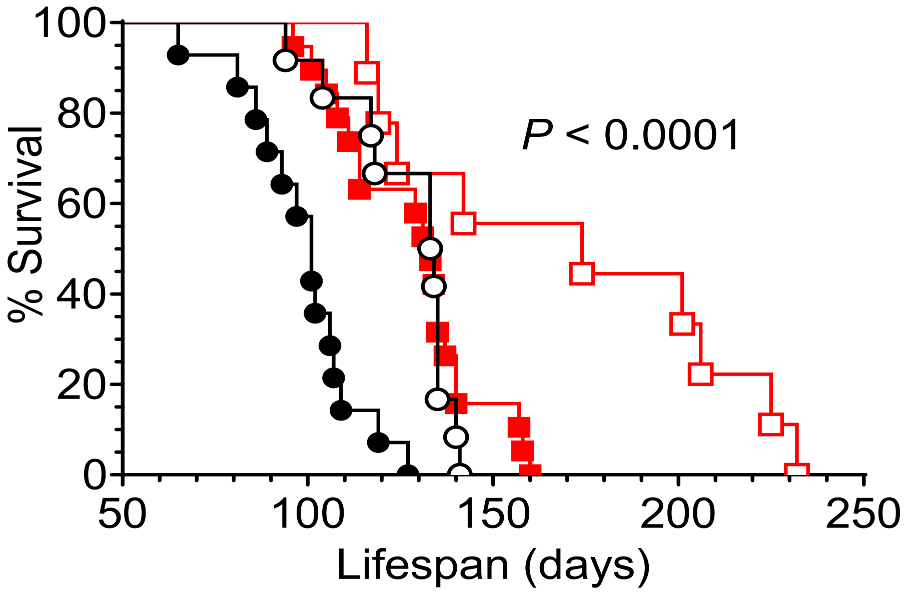

Subsets of sICH Dahl S rats		Median Survival
● F, tg+	[n = 14]	101 days
○ F, wt	[n = 12]	133 days
■ M, tg+	[n = 19]	133 days
□ M, wt	[n = 9]	174 days

Supplementary Fig. S2. Representative *ex vivo* MRI of sICH rat brains

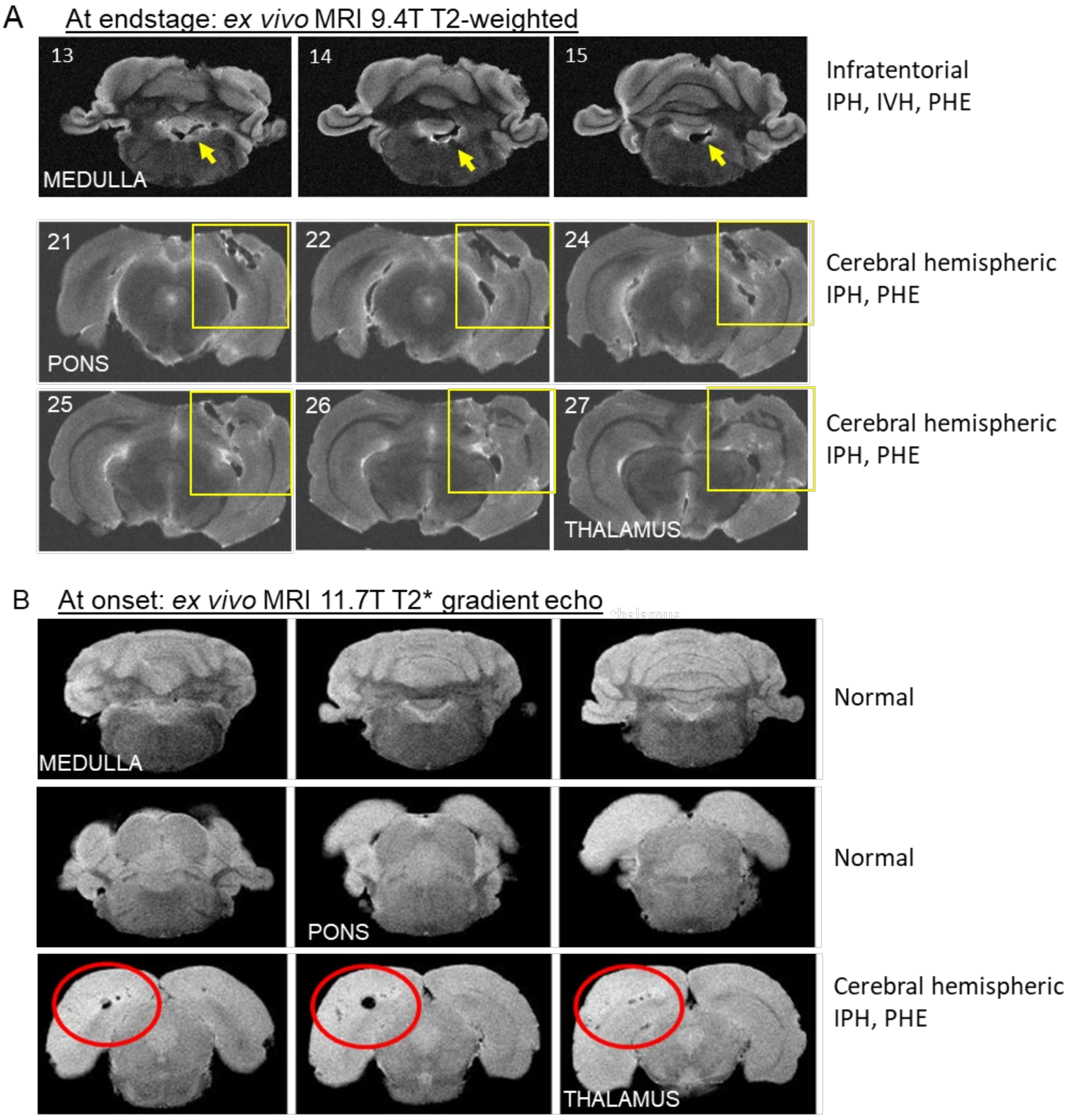

Supplementary Fig. S3. Binding curves of anti-DEspR antibodies 10a3 (rat-specific) and 6g8 (human-rat cross reactive)

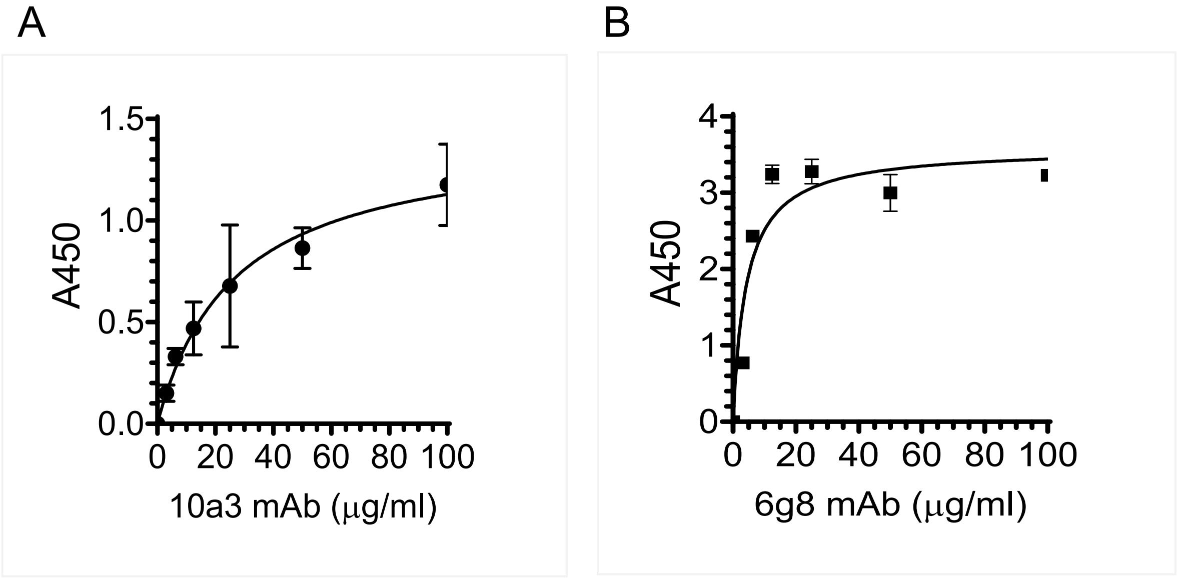

Supplementary Fig. S4. Age at sICH detection in study groups: treated (Tx) and non-treated (nonTx) female (F) and male (M) rats

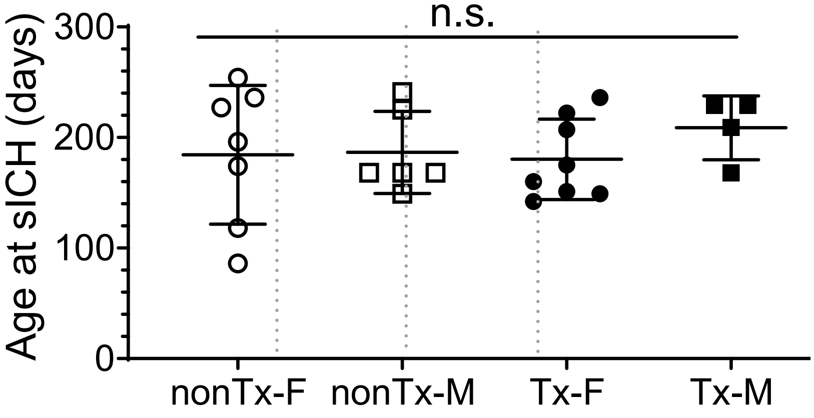

Supplement: Supplementary file 1 — Supplementary Information 1. [file 41598_2023_28149_MOESM1_ESM.pdf]
