# Supplementary material for: Anti-DEspR antibody treatment improves survival and reduces neurologic deficits in a hypertensive, spontaneous intracerebral hemorrhage (hsICH) rat model
Source: Sci Rep. 2023 Feb 15;13:2703. doi: 10.1038/s41598-023-28149-3 (PMC9932093; doi:10.1038/s41598-023-28149-3)
Supplement: Supplementary file 2 — Supplementary Information 2. [file 41598_2023_28149_MOESM2_ESM.pdf]

Supplementary Table S1. Characteristics of hu6g8(single i.v. 1 mg/kg/dose)-treated rats after monitoring to ascertain non-transient neurological deficits prior to treatment

ID	Sex	Geno-type	Age at sICH	NDS	Neurological deficits prior to treatment	Time to improvement
52	F	tg	175 d	3	Spasticity, paresis, increasing seizure frequency then decreased LOC	24 hrs
63	F	wt	222 d	3	Left sided paresis, decreased coordination, slightly decreased LOC	24 hrs *ex vivo MRI*
64	F	wt	236 d	3	Decreased movement, head bobbing, focal seizures, increased rigidity	48 hrs
49	F	wt	207 d	4	Right lower limb paresis, spasticity, decreased coordination, upper limbs, bruxism, → decreased LOC	48 hrs
51	F	wt	142 d	5	Ataxia, increasing myoclonic seizures, decorticate posturing, →decreased LOC	44 hrs
38	F	tg	149 d	5	Myoclonic seizure involving face, neck, right forelimb, paresis of right hind limb, facial weakness, → decreased LOC	54 hrs
56	F	tg	151 d	5	Full left sided paresis, spasticity on right side, decreased LOC, spasticity of LE, seizures; progression to upper limb paralysis, lower limb restored	Died
57	F	tg	160 d	5	Eye twitch, spasticity, facial twitch, → full body paresis, decreased LOC	Died
45	M	tg	168 d	1	Myoclonic jerks (body/ head), right facial weakness	24 hrs
43	M	tg	229 d	2	Right upper limb, paresis, increased respiratory rate, decreased coordination, slowed response, spasticity	48 hrs *ex vivo MRI*
55	M	wt	209 d	5	Decreased coordination, upper limb paresis > lower; right > left, →decreased LOC	24 hrs
42	M	wt	229 d	5	Decreased coordination, increased spasticity, →decreased LOC	48 hrs

Legend: >, greater than; →, progress to…; d, days; *ex vivo* MRI, post-mortem MRI confirms sICH diagnosis with intraparenchymal hemorrhage, perihematomal edema and intraventricular hemorrhage to varying degrees; F, female; hrs, hours from treatment; LOC, level of consciousness; M, male; NDS, neurological deficit scale based on rat-adapted mRS; tg, transgenic (hyperlipidemic) sICH-prone Dahl Salt sensitive rat; wt, wild type (normolipidemic) sICH-prone Dahl salt-sensitive rat.

Supplementary Table S2. Littermate control sICH rats.

ID	Sex	Geno-type	Age at sICH	Neurologic deficit score at last check		Post-mortem confirm'n and *ex vivo* MRI
58	F	wt	86 d	Last check ≤ 12 hrs prior: < 3 NDS, no euthanasia indicators	Spontaneous death	☑
59	F	tg	118 d	Last check ≤ 16 hrs prior: < 3 NDS, no euthanasia indicators	Spontaneous death	☑
53	F	wt	174 d	Last check ≤ 16 hrs prior: < 3 NDS, no euthanasia indicators	Spontaneous death	☑ *ex vivo* MRI
70	F	tg	196 d	Last check ≤ 16 hrs prior: < 3 NDS, no euthanasia indicators	Spontaneous death	☑
48	F	tg	227 d	Last check ≤ 16 hrs prior: < 3 NDS, no euthanasia indicators	Spontaneous death	☑ *ex vivo* MRI
62	F	wt	236 d	Last check ≤ 16 hrs prior: < 3 NDS, no euthanasia indicators	Spontaneous death	☑ *ex vivo* MRI
46	M	wt	149 d	Last check ≤ 16 hrs prior: < 3 NDS, no euthanasia indicators	Spontaneous death	☑
74	M	tg	168 d	NDS-5, euthanasia indicators +	Euthanized	☑ *ex vivo* MRI
44	M	tg	168 d	Last check ≤ 16 hrs prior: < 3 NDS, no euthanasia indicators	Spontaneous death	☑ *ex vivo* MRI
66	M	tg	168 d	Last check ≤ 16 hrs prior: < 3 NDS, no euthanasia indicators	Spontaneous death	☑
54	M	tg	225 d	Last check ≤ 16 hrs prior: < 3 NDS, no euthanasia indicators	Spontaneous death	☑
67	M	tg	241 d	Last check ≤ 16 hrs prior: < 3 NDS, no euthanasia indicators	Spontaneous death	☑ *ex vivo* MRI

Legend: >, greater than; →, progress to…; d, days; *ex vivo* MRI, post-mortem MRI confirms sICH diagnosis with intraparenchymal hemorrhage, perihematomal edema and intraventricular hemorrhage to varying degrees; F, female; hrs, hours from treatment; LOC, level of consciousness; M, male; NDS, neurological deficit scale based on rat-adapted mRS; tg, transgenic (hyperlipidemic) sICH-prone Dahl Salt sensitive rat; wt, wild type (normolipidemic) sICH-prone Dahl salt-sensitive rat.
