# Supplementary material for: Anti-DEspR antibody treatment improves survival and reduces neurologic deficits in a hypertensive, spontaneous intracerebral hemorrhage (hsICH) rat model
Source: Sci Rep. 2023 Feb 15;13:2703. doi: 10.1038/s41598-023-28149-3 (PMC9932093; doi:10.1038/s41598-023-28149-3)
Supplement: Supplementary file 3 — Supplementary Information 3. [file 41598_2023_28149_MOESM3_ESM.pdf]

Supplementary Figure Legends

Supplementary Fig. S1. Kaplan-Meier survival curves of sICH rat model subsets based on sex and presence/absence of transgenic hCETP-mediated hyperlipidemia.[1] Female (F) and male (M) transgenic (tg+) hyperlidemic rats (median survival 101 days and 133 days respectively), female (F) and male (M) non-transgenic or wild type (wt) normolipidemic rats (median survival 133 and 174 days respectively. Mantel-Cox log rank test for significance, $P < 0.0001$.

Supplementary Fig. S2. Representative *ex vivo* MR-images of sICH rat model brain obtained at end stage and at onset of ICH. (A) End-stage sICH rat brain: 9.4T MRI T2-weighted images at the level of the medulla (#13-15); pons (#21-#24), and thalamus (#27). Right side on MRI = rat left; 52 slices: #1 caudal-rostral #52. Yellow ➔ points to IVH in the 4th ventricle, IPH and PHE in surrounding area. Yellow box highlights area with IPH and PHE. (B) 11.7T MR T2*-weighted gradient echo images at the same planes shown in A, and contrasted to extent of IPH and PHE at end-stage lesions.

Supplementary Fig. S3. Characterization of ratDEspR-reactive murine monoclonal antibodies (mAb): 10a3 and 6g8, precursor to the humanized human-rat-monkey reactive mAb, hu6g8. (A) Saturation binding curve of 10a3 mAb, with a murine IgG1 backbone, to its antigenic peptide on the extracellular domain of rat DEspR: PLLTSLGSKE (rat-specific). ELISA performed in duplicates for each point, EC50 26.5 ± 4,2 μg/ml binding affinity. (B) Saturation binding curve of 6g8 mAb, with a murine IgG2b Fc backbone, to its antigenic peptide on the extracellular predicted binding domain of DEspR: EMKSRWNWGS (rat, human, monkey cross-reactive). ELISA was performed in duplicates for each point: EC50 4.3 ± 2.1 μg/ml binding affinity. 6g8 was selected for humanization with a human hinge-stabilized IgG4 backbone.

Supplementary Fig. S4. Age at sICH onset assigned to hu6g8 treated and non-treated study groups. Comparison of mean age at sICH onset among untreated (nonTx) controls and treated (Tx) male (M) and female (F) sICH rats: group means are not significantly (ns) different (One-way ANOVA). Control non-treated (nonTx, n = 13): 7 female, 6 male hsICH-rats; Treated (Tx, n = 12): 8 female, 4 male hsICH-rats.

---

[1] Decano, J.L. *et al*. Early-life sodium exposure unmasks susceptibility to stroke in hyperlipidemic, hypertensive heterozygous Tg25 rats transgenic for human cholesteryl ester transfer protein. *Circulation* **119**, 1501-1509. doi: 10.1161/CIRCULATIONAHA.108.833327 (2009).
